# Identification of Twelve Different Mineral Deficiencies in Hydroponically Grown Sunflower Plants on the Basis of Short Measurements of the Fluorescence and P700 Oxidation/Reduction Kinetics

**DOI:** 10.3389/fpls.2022.894607

**Published:** 2022-06-02

**Authors:** Gert Schansker, Miho Ohnishi, Riu Furutani, Chikahiro Miyake

**Affiliations:** ^1^Heinz Walz GmbH, Effeltrich, Germany; ^2^Department of Applied Biological Science, Graduate School for Agricultural Science, Kobe University, Kobe, Japan

**Keywords:** mineral deficiencies, P700-kinetics, polyphasic fluorescence rise, signatures, photosynthetic electron transport chain

## Abstract

The photosynthetic electron transport chain is mineral rich. Specific mineral deficiencies can modify the electron transport chain specifically. Here, it is shown that on the basis of 2 short Chl fluorescence and P700^+^ measurements (approx. 1 s each), it is possible to discriminate between 10 out of 12 different mineral deficiencies: B, Ca, Cu, Fe, K, Mg, Mn, Mo, N, P, S, and Zn. B- and Mo-deficient plants require somewhat longer measurements to detect the feedback inhibition they induce. Eight out of twelve deficiencies mainly affect PS I and NIR measurements are, therefore, very important for this analysis. In Cu- and P-deficient plants, electron flow from the plastoquinone pool to PS I, is affected. In the case of Cu-deficiency due to the loss of plastocyanin and in the case of P-deficiency probably due to a fast and strong generation of Photosynthetic Control. For several Ca-, K-, and Zn-deficient plant species, higher levels of reactive oxygen species have been measured in the literature. Here, it is shown that this not only leads to a loss of Pm (maximum P700 redox change) reflecting a lower PS I content, but also to much faster P700^+^ re-reduction kinetics during the I_2_-P (~30–200 ms) fluorescence rise phase. The different mineral deficiencies affect the relation between the I_2_-P and P700^+^ kinetics in different ways and this is used to discuss the nature of the relationship between these two parameters.

## Introduction

For optimal plant growth, a balanced diet of minerals is needed. If there is an insufficient supply of a particular mineral, this may lead to crop losses. It is possible to determine the mineral status of the soil by analyzing soil samples, however, for detection of mineral deficiencies, the plants growing on this soil can also be used. Since many of these mineral deficiencies directly or indirectly affect the photosynthetic apparatus and its activity, non-invasive methods like chlorophyll (Chl) *a* fluorescence and near-infrared (NIR) absorption measurements have the potential to identify and detect the different mineral deficiencies.

Since the introduction of instruments for the measurement of Chl fluorescence and NIR kinetics (820 nm single channel measurements, P700 measurements derived from the difference between 830 and 870 nm, and deconvolution of PC, P700, and Fd signals on the basis of the measurement of 4 NIR wavelength pairs), it is possible to study the kinetics of photosystem (PS) II and PS I in detail. In a recent study, Ohnishi et al. (2021) characterized twelve mineral deficiencies on the basis of the kinetics of the operational quantum yield of PS II: Y(II), operational quantum yield of PS I: Y(I), yield of PS I donor side limitations: Y(ND), and yield of PS I acceptor side limitations: Y(NA) measured during 10 min of illumination, using a DUAL-PAM-100. This approach gives information on the impact of these 12 deficiencies on photosynthetic activity. For this study, also classical saturation pulses (300 ms high red-light intensity) and pulses for Pm-determinations (to determine the difference between all P700 oxidized and reduced) were applied to these plants. Here, we took these kinetic measurements and analyzed the polyphasic fluorescence rise and P700-kinetics during the first second of illumination focusing on lesions in the photosynthetic electron transport chain induced by these 12 deficiencies. Measurements of the polyphasic fluorescence rise and the simultaneously measured P700 kinetics give information on the reduction of the electron transport chain (Schansker et al., 2005). For the interpretation of the mineral deficiency data, we made use of the effect of the destruction of the manganese cluster (Tóth et al., 2007a), the effects of Mg and S-deficiency (Ceppi et al., 2012; Dinç et al., 2012), and the effects of salt stress (Çiçek et al., 2018) on the chlorophyll and 820 nm kinetics, considering as well, the relationship between the fluorescence kinetics and the plastoquinone (PQ) pool redox state (Tóth et al., 2007b).

To help the interpretation of the data, first an analysis of the literature was made. Ohnishi et al. (2021) worked with sunflower plants that were grown hydroponically. The following mineral deficiencies were induced: B-, Ca-, Cu-, Fe-, K-, Mg-, Mn-, Mo-, N-, P-, S-, and Zn-deficiency. What we want to show in this paper is, that short measurements of the Chl fluorescence and P700 kinetics of about 1 s contain enough information to identify specific signatures for each of these 12 mineral deficiencies.

## Materials and Methods

### Plant Material

Sunflower (*Helianthus annuus*) plants were grown in a growth chamber (14 h light at 27°C/10 h dark at 25°C; light intensity 300–400 μMol photons m^−2^ s^−1^; relative humidity 50–60%). Seeds of sunflower were germinated in flowing tap water for 5 days in the growth chamber. Five-day-old seedlings were planted in pots filled with a hydroponic solution of half-strength Hoagland solution for 3 days. The plants were then transferred to full-strength Hoagland solution for 4 days. The composition of full-strength Hoagland solution is as follows: macronutrients [NH_4_NO_3_ (2 mM), KH_2_PO_4_ (0.8 mM), CaCl_2_ (0.6 mM), and MgSO_4_ (0.5 mM)]; micronutrients [H_3_BO_4_ (50 μM), MnSO_4_ (9 μM), ZnSO_4_ (0.7 μM), CuSO_4_ (0.3 μM), Na_2_MoO_4_ (0.25 μM), and NaFeEDTA (45 μM)]. The pH of the nutrient solution was adjusted to 5.3–5.5, by adding 10 mM MES (2-morpholinoethanesulfonic acid) during cultivation. The plants were then transferred to the original Hoagland solution deprived of essential minerals (N, P, K, S, Ca, Zn, Mo, B, Fe, Mn, Cu, and Mg) for 1 week. Then, in the 1 week period after the 1 week of exposure to the treatment, the measurements of each sample were taken. The solutions were renewed once a week and always aerated with air. For all experiments, we measured the second leaves from the top of the plants.

### Measurements of Fast PSI and PSII Kinetics

P700^+^ absorbance and chlorophyll fluorescence were simultaneously measured using a Dual-PAM/F instrument (Walz, Effeltrich, Germany) with a closed leaf-type chamber (Bunkoukeiki Co., Ltd., Tokyo, Japan), in which expiratory air (assumed to be CO_2_ saturated air) was let in and the temperature was set at 25 ± 0.1°C using a Peltier controller system (Bunkoukeiki Co., Ltd. Tokyo, Japan). Leaf disks (3.4 cm^2^) from dark-adapted sunflowers were placed in the chamber.

Two types of kinetic measurements were analyzed here. Saturation pulse (SP) measurements consisting of 630 nm light pulses of 300 ms (16,000 μMol photons m^−2^ s^−1^). For the determination of Pm, SP illumination was applied after 10 s of illumination with far-red light (720 nm).

## What Does the Literature Tell Us About These Mineral Deficiencies?

### Cu-Deficiency

For the green alga *Chlamydomonas,* it was shown that in the case of Cu-deficiency, the copper containing plastocyanin is replaced by the iron-containing cytochrome *c*_6_ as reviewed by Merchant et al. (2006). There are few studies on the physiological effects of copper deficiency on vascular plants, but also in plants plastocyanin would be a potential target. Ohnishi et al. (2021) indeed observed on the basis of DUAL-KLAS-NIR measurements, allowing the quantification of the PC signal, that Cu-deficient plants have a much lower PC/P700 ratio. Another potential target is the Cu/Zn superoxide dismutase (SOD; Tainer et al., 1983; Bowler et al., 1992). Yamasaki et al. (2008) note that at low Cu-concentrations Cu/Zn-SOD is replaced by Fe-SOD in *Arabidopsis*.

### P-Deficiency

Frydenvang et al. (2015) demonstrated for barley that P-deficiency made the I_2_-step of O-I_1_-I_2_-P transient disappear. A similar effect was observed by Cetner et al. (2020) for radish plants. Frydenvang et al. (2015) could predict the severity of the treatment by quantifying this effect. In a subsequent paper (Carstensen et al., 2018), it was shown that P-deficiency effects were rapidly reversible. The authors showed as well that P-deficiency strongly reduced the orthophosphate content in the stroma. The authors concluded that P-deficiency leads to an inhibition of ATP-synthase. Looking at the electron transport chain, this could lead to a rapid build-up of Photosynthetic Control and, thus, slowdown of electron transfer from the PQ-pool to P700.

### Mn-Deficiency

Husted et al. (2009) showed for Mn-deficient barley plants that their O-I_1_-I_2_-P transients behaved like transients of plants that had been exposed to a heat pulse in darkness (Srivastava et al., 1997; Tóth et al., 2005, 2007a) leading to a loss of their Mn-cluster. The authors write about genotypic differences between two barley varieties, but those differences look more like differences in the tolerance of the two varieties used to Mn-deficiency, with the variety Vanessa being considerably more tolerant than the variety Antonia. The differences in State Transitions observed by the authors may also be due to differences in the PS II electron donation capacity (depending on the severity of the deficiency) and associated differences in the PQ-pool redox state. In a follow-up paper, Birkelund Schmidt et al. (2013) showed that below a Mn-concentration of ~11 mg Mn kg^−1^ plant dry weight, the apparent F_V_/F_M_ value correlated well with the Mn-content of the barley plants. In a third paper, the effects of the severity of the Mn-deficiency stress and recovery on the parameters of the quenching analysis (Ф_PSII_, qP, and NPQ) were studied (Schmidt et al., 2016).

### Fe- and S-Deficiencies

At the acceptor side of PS I, a high concentration of FeS-clusters is found of which three belong to PS I and ferredoxin also contains an FeS-cluster. Further, two FeS-clusters are found in the Rieske protein of the cytochrome *b*_6_*f* complex and there is a non-heme iron in PS II located between Q_A_ and Q_B_. Spiller and Terry (1980) studied the development of Fe-deficiency-related changes in “fairly” mature sugar beet leaves. Fe-deficiency developed within 10 days; this is considerably faster than the development of S-deficiency-related changes in another study on sugar beet leaves (Ceppi et al., 2012). This may also explain the fact that the Fe-deficiency-related changes observed here were considerably more severe than the S-deficiency-related changes. Stiller and Terry observed that, initially, P700 and cyt *f* content decreased in parallel and was also paralleled by the loss of chlorophyll. The authors concluded that the plants lost electron transport chains, reflected by a reduction in the number of granal and stromal lamellae per chloroplast, but the photosynthetic unit size of the remaining electron transport chains was not affected. Only under severe stress conditions, the loss of P700 was considerably higher than the loss of cyt *f* and chlorophyll.

S-deficient sugar beet plants are also characterized by loss of the P700 + PC signal (Ceppi et al., 2012). There it was shown that for sugar beet leaves, the loss of the 820-nm signal correlated quite well with a decrease in the I_2_-P amplitude.

### Ca-, K-, and Zn-Deficiencies

Terry and Huston (1975) studied calcium deficiency in hydroponically grown sugar beet plants and found little effect of calcium deficiency on processes ranging from CO_2_ assimilation to electron transport activities. Ridolfi et al. (1996) observed for Ca-deficient oak that it also caused a decrease of the Mg-content. In oak, Ca-deficiency had little effect on steady state fluorescence parameters, but Ca-deficient oak leaves showed more photorespiration. De Freitas et al. (2016) suggested a relationship between Ca-deficiency, gibberellic acid, and ROS scavenging capacity. Although no solid experimental proof for this suggestion was provided, it fits best with the experimental observations obtained for the sunflower plants studied here.

Also, K-deficiency has been associated with increased ROS production. Cakmak (2005) noted in a review that evidence is accumulating that improvement of the potassium nutritional status of plants can greatly lower the ROS production. Though many factors related to K-deficiency have been described in the literature the exact mechanism by which K-deficiency negatively affects photosynthesis remains unclear.

As noted above, one of the superoxide dismutases is a Cu/Zn superoxide dismutase. This enzyme may be a target for Zn-deficiency. And this could then lead to higher levels of ROS, damage to the acceptor side of PS I and a loss of Pm-amplitude.

Based on the literature Mg- and N-deficiency also have been associated with higher levels of ROS, but these deficiencies also induce additional deficiency-related changes and will, therefore, be discussed separately (see below).

### Mg-Deficiency

An early response of plants to Mg-deficiency is a strong accumulation of sugar and starch (Fischer and Bremer, 1993; Hermans et al., 2005; Cakmak and Kirkby, 2008). Mg-deficiency also leads to a light intensity-dependent loss of chlorophyll (Cakmak and Kirkby, 2008; Peng et al., 2019), which does not lead to a change in the chl *a*/*b* ratio, nor does it affect the functionality of the remaining electron transport chains (Terry and Ulrich, 1974; Dinç et al., 2012). Another symptom of Mg-deficiency is the destacking of thylakoid membranes as, for example, observed in maize (Hall et al., 1972). For sugar beet leaves, it was suggested that destacking and an associated increase in spillover could be responsible for some of the observed phenomena (Dinç et al., 2012). The literature further suggests that Mg-deficiency in a late stage increases ROS levels and may, therefore, cause damage to the PS I acceptor side with an associated loss of the Pm-amplitude (Cai et al., 2019; Hauer-Jákli and Tränkner, 2019; Peng et al., 2019).

### B- and Mo-Deficiencies

Boron deficiency leads to an accumulation of sugar in olive leaves (Liakopoulos et al., 2005). For *Citrus sinensis,* both increased leaf levels of starch and hexoses (Han et al., 2008) and an accumulation of phenolics and a decreased nitrate reductase activity have been described (Lu et al., 2014). For *Brassica rapa* leaves, starch accumulation was reported (Hajiboland and Farhanghi, 2010). Feedback inhibition of photosynthesis due to the accumulation of synthates has been suggested (e.g., Dell and Huang, 1997).

With respect to Mo-deficiency, there are quite a few articles that describe in detail all enzymes that contain Mo as a cofactor. However, the impact on the activity of the electron transport chain is rarely described. Molybdenum is a cofactor of nitrate reductase. Mahler (1982) writes that Mo-deficient non-legume plants cannot make use of nitrate taken up by the roots. As a consequence, Mo-deficiency induces N-deficiency and in the field, it is difficult to discriminate between Mo- and N-deficiency. Ide et al. (2011) analyzed the metabolic profile of Mo-deficient plants and noted a decrease in amino acids and an increase in sugars and sugar phosphates related to an increase in the carbon to nitrogen ratio. The increased sugar levels could lead to feedback inhibition.

### N-Deficiency

Evans and Terashima (1988) concluded that N-deficiency had no effect on photosynthetic activities when calculated on a chlorophyll basis. Oxidative stress has been described though. Shao et al. (2020) observed for rice that N-deficiency leads to increased H_2_O_2_ production and lipid peroxidation. Nguyen et al. (2003) found for *Melaleuca* and *Eucalyptus* species grown under N-deficient conditions an accumulation of starch and sugars in different parts of these plants, also in the leaves, suggesting that N-deficiency may lead to feedback inhibition.

## Experimental Observations Derived From the Dataset

**Figure 1:**
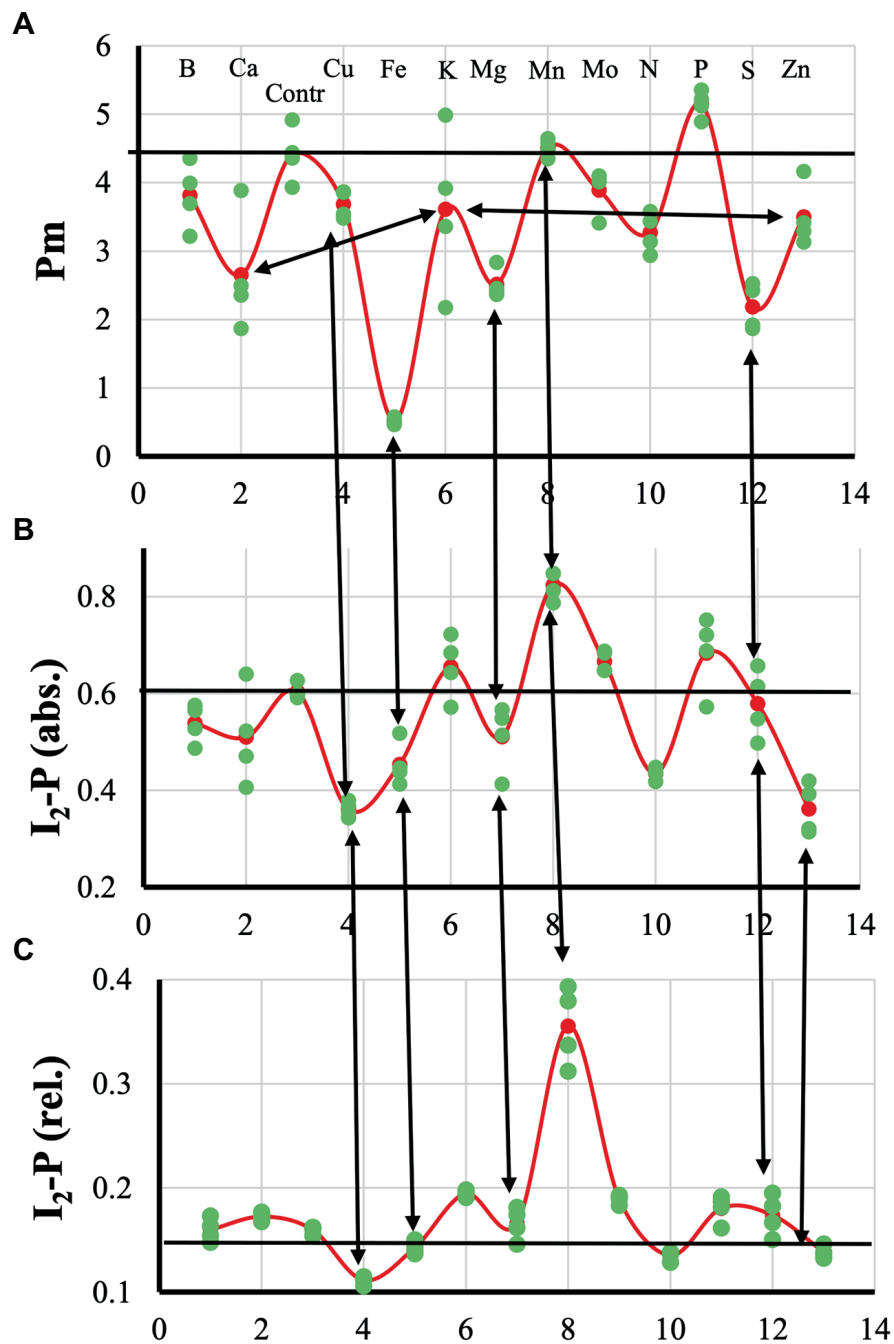
Effect of the 12 mineral deficiencies applied to hydroponically grown sunflower plants on the Pm **(A)**, absolute **(B)**, and relative I_2_-P **(C)** amplitudes, respectively.

Figure 1 gives an overview of the effects of the different mineral deficiencies on the Pm (Figure 1A), the absolute (Figure 1B) and relative I_2_-P amplitude (Figure 1C). In Supplementary Figure 1, all averages were normalized to the control values. These data will be discussed in the context of the different deficiencies.

**Figure 2:**
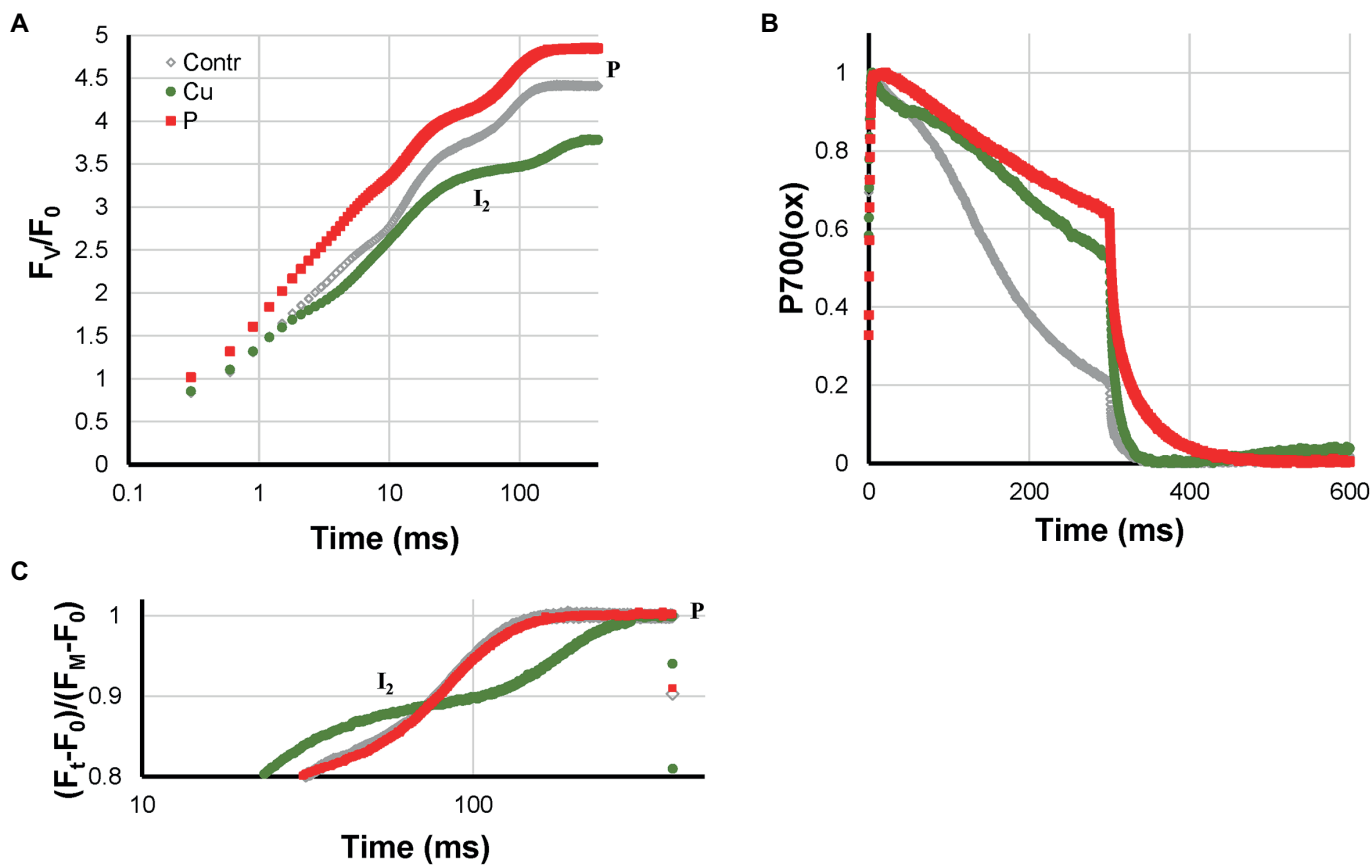
Effect of P- and Cu-deficiencies applied to hydroponically grown sunflower plants on the polyphasic rise **(A)** and P700^+^ kinetics **(B)**, respectively. In panel **(C)**, the double-normalized I_2_-P amplitudes are shown to allow a comparison of the rise kinetics. The transients are averages of four independent measurements.

**Figure 3:**
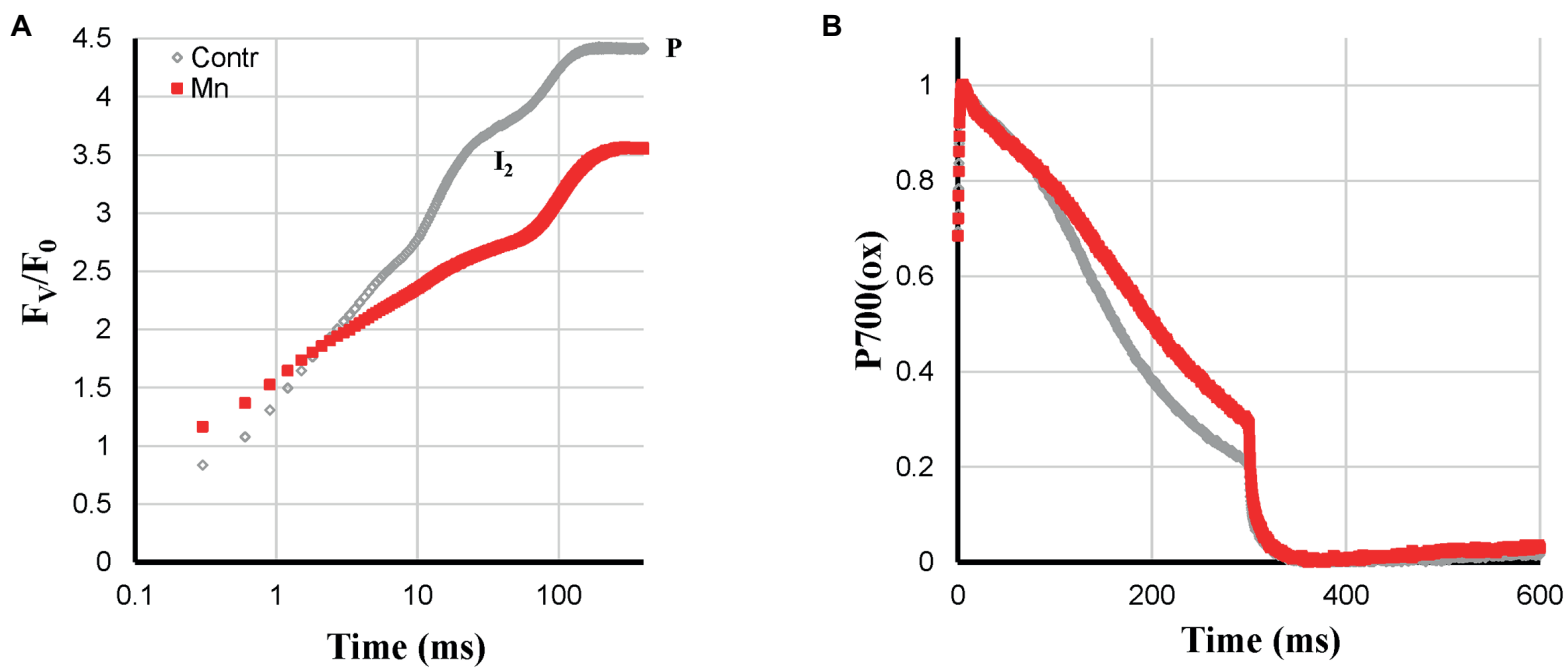
Effect of Mn-deficiency applied to hydroponically grown sunflower plants on the polyphasic rise **(A)** and P700^+^ **(B)** kinetics, respectively. The transients are averages of four independent measurements.

**Figure 4:**
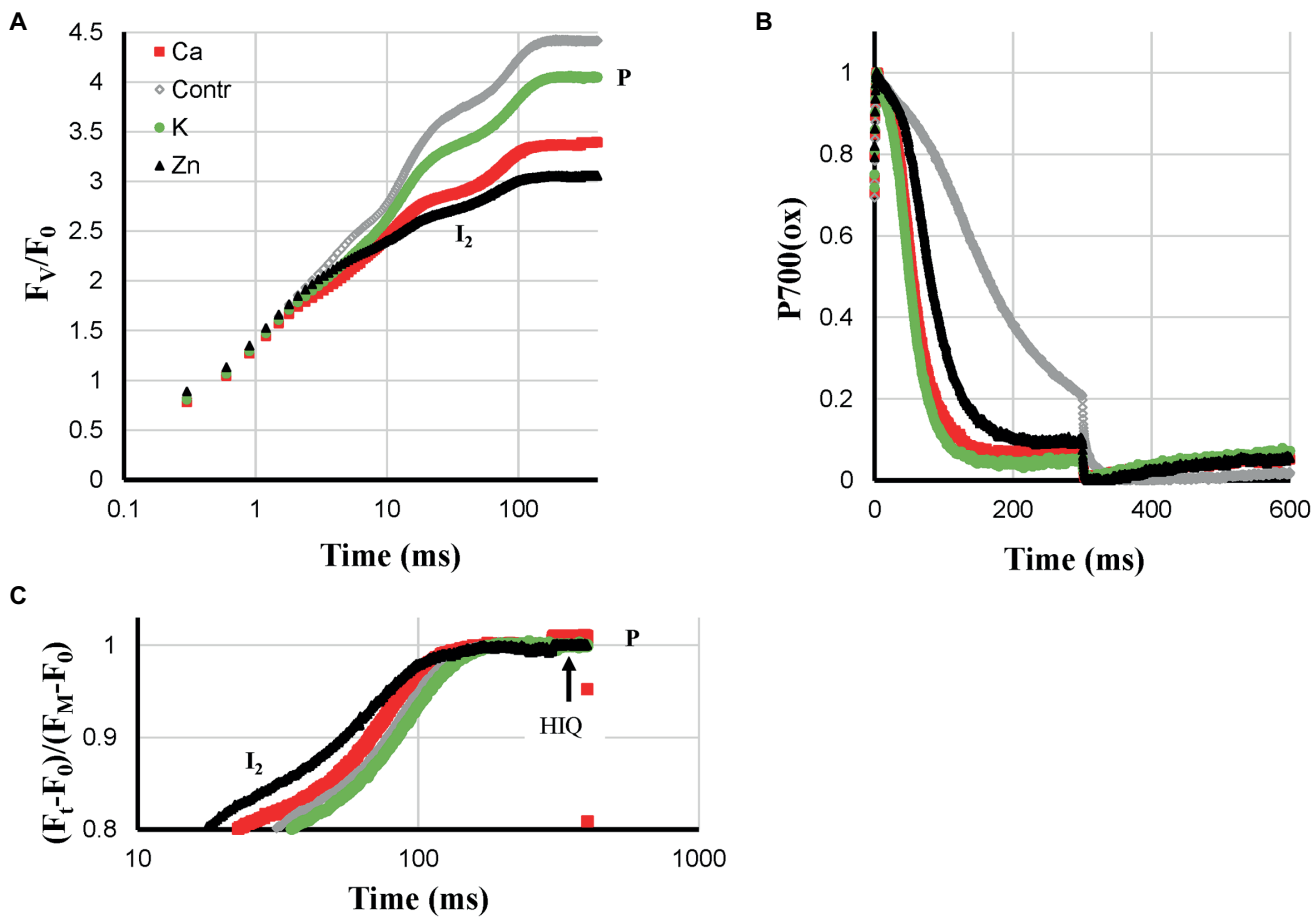
Effect of Ca-, K- and Zn-deficiencies applied to hydroponically grown sunflower plants on the polyphasic rise **(A)** and P700^+^ **(B)** kinetics, respectively. In panel **(C)** the double-normalized I_2_-P amplitudes are shown to allow a comparison of the rise kinetics. The transients are averages of four independent measurements.

Cu- and P-deficiency both affected the electron flow from the PQ-pool to P700. In both cases, the re-reduction of P700^+^ during the I_2_-P phase was very slow. At the P-level, in both cases, around 60% of P700 remained in the oxidized state (Figure 2B). Differences between the two are that P-deficient plants had an F_V_/F_0_ that was considerably higher than the Control and no HIQ (high intensity quenching, lowering of the fluorescence intensity due to the formation of fluorescence quenching carotenoid radicals in the light; Schreiber et al., 2019); Cu-deficient plants had an F_V_/F_0_ value that was considerable smaller than the Control and a little bit of HIQ (in Figures 3, 4 the position of the more prominent HIQ is indicated with an arrow). Further, the I_2_-P kinetics of P-deficient and Control plants were very similar, whereas, in the case of Cu-deficiency, slower kinetics and a considerable lag-phase were observed (Figures 2A,C). In addition, the P700^+^ re-reduction kinetics were more complex in the case of Cu-deficient plants and the effect of the deficiency on the fraction loss of I_2_-P (abs.) and I_2_-P (rel.) was stronger than the fraction Pm loss (see Supplementary Figure 1).

Mn-deficiency was expected to lead to a gradual loss of Mn-clusters of the PS II reaction centers. It was, therefore, expected that Mn-deficient plants behaved like heat-pulse-treated plants, which have a very limited PS II electron donation capacity. This was indeed observed as: slow reduction kinetics of the electron transport chain, by far the largest I_2_-P amplitude and an associated quite strong reduction of F_V_/F_0_ (Figure 3A). The P700^+^ re-reduction kinetics on the other hand were not too different. The re-reduction of the Mn-deficient leaves was less complete, which may be related to the lower electron donation capacity of PS II (Figure 3B). Compared to the Mn-deficiency-related changes of the barley variety Antonia described by Husted et al. (2009), the effect of the treatment on these sunflower plants was still rather mild.

For Ca-, K-, and Zn-deficiencies, higher ROS levels have been described and these three deficiencies led to a loss of Pm: Ca > K > Zn. Ca- and K-deficient plants both showed very fast P700^+^ re-reduction kinetics during the I_2_-P phase. These kinetics were somewhat slower in Zn-deficient plants but still much faster than in the Control plants (Figure 4B). HIQ was present but not very strong in Ca-deficient plants, very small in Zn-deficient plants, and absent in K-deficient plants. Further, Ca- and Zn-deficient plants showed quite low F_V_/F_0_ values of approx. 3.35 and 3.05, respectively, compared with approx. 4.05 for K-deficient plants (Figures 4A,C). In Figure 3C, the I_2_-P rise kinetics of fluorescence transients double-normalized between O and P are shown, indicating that Zn-deficient plants had the fastest rise time followed by Ca-deficient plants. The rise times observed for the Control and K-deficient plants are similar.

**Figure 5:**
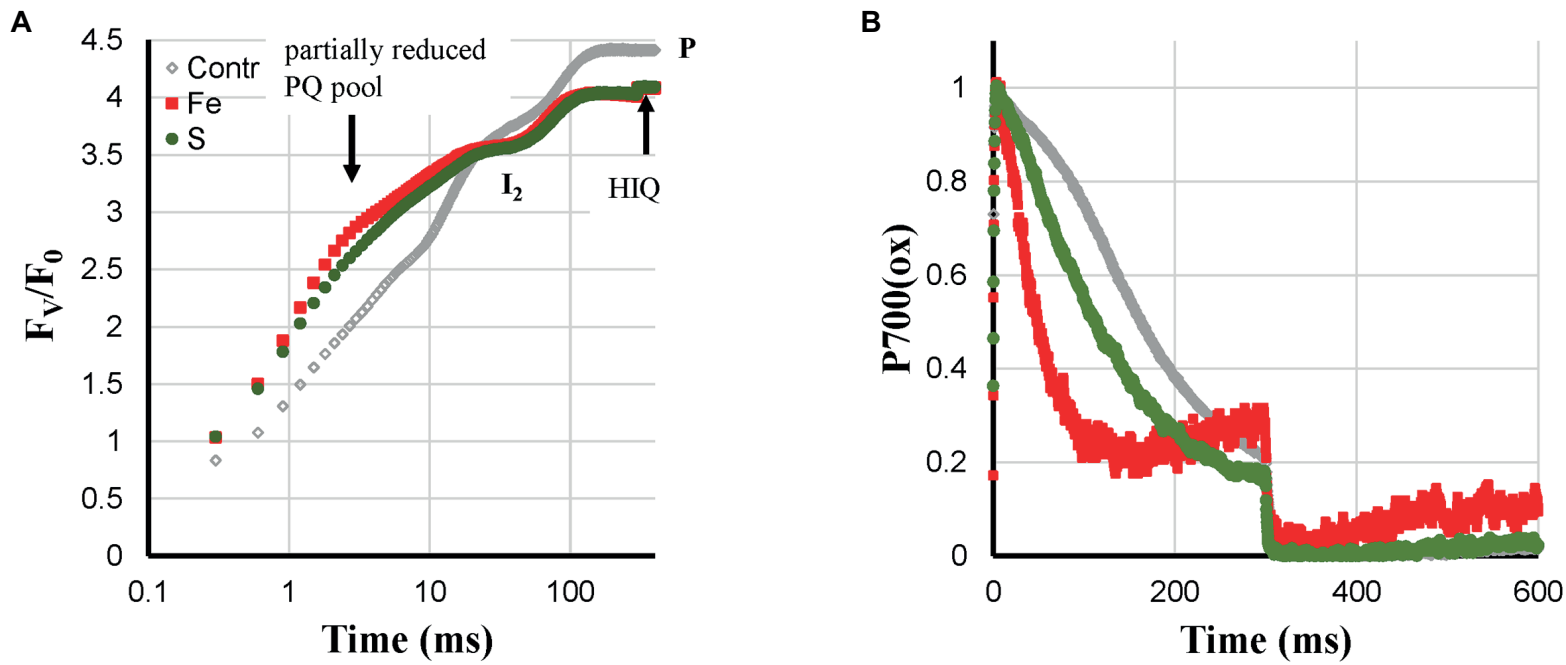
Effect of Fe- and S-deficiencies applied to hydroponically grown sunflower plants on the polyphasic rise **(A)** and P700+ **(B)** kinetics, respectively.

Fe- and S-deficient plants were expected to synthesize fewer FeS-clusters and as a consequence have a lower PS I content. These two deficiencies showed the strongest loss of Pm; however, the development of deficiency-related changes was considerably faster and stronger in Fe-deficient plants than in S-deficient plants. Fe-deficiency led to faster P700^+^ re-reduction kinetics during the I_2_-P rise, a symptom that was not pronounced in the S-deficient leaves (Figure 5B). The fast P700^+^ re-reduction kinetics described for Ca-, K-, and Zn-deficiencies described above may also be explained by a ROS-induced damage to the FeS-clusters of the PS I acceptor side. Higher ROS levels due to a loss of the Fe-SOD could be an explanation for the fast P700^+^ re-reduction kinetics observed for the Fe-deficient plants that is missing in the case of S-deficient plants. Despite the difference in Pm-amplitude (88% loss in Fe-deficient leaves compared with 50% loss in S-deficient leaves), the induction curves of Fe- and S-deficient plants were quite similar. Both curves showed a partially reduced PQ-pool (Tóth et al., 2007b), an F_V_/F_0_-value of about 4 and HIQ (Figure 5A). The partially reduced PQ-pool in darkness was also observed under quite severe S-deficient conditions in sugar beet (Dinç et al., 2012). This was not observed for Ca-, K-, N-, and Zn-deficient plants, where the smaller Pm may be due to ROS-induced destruction of the PS I acceptor side.

**Figure 6:**
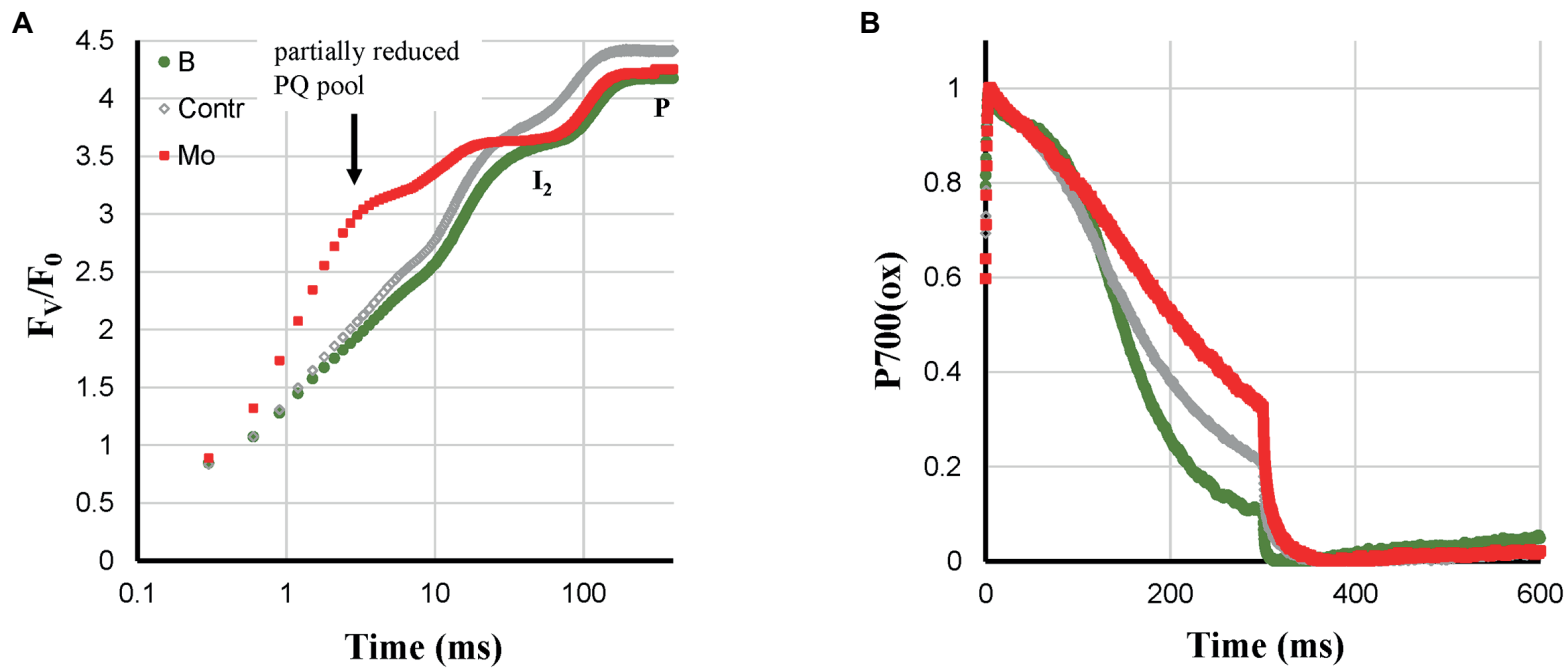
Effect of B- and Mo-deficiencies applied to hydroponically grown sunflower plants on the polyphasic rise **(A)** and P700^+^ **(B)** kinetics, respectively. The transients are averages of four independent measurements.

Based on the literature, Mo- and B-deficiency could lead to feedback inhibition (see above). Mo- and B-deficiency had no effect on Pm and showed only a small decrease of F_V_/F_0_ (Figure 6A). Mo-deficient plants showed a strong reduction of the PQ-pool in darkness with an associated broad I_2_-step (Tóth et al., 2007b). Further, Mo-deficient plants showed an incomplete re-reduction of P700^+^ (Figure 6B) and HIQ. B-deficient plants had more sigmoidal P700^+^ re-reduction kinetics than the Control (Figure 6B) and showed no HIQ. However, these two types of plants are better defined by an almost non-existent P700-donor side limitation Y(ND) and a high PS I acceptor side limitation Y(NA) during most of a 10 min induction period (Ohnishi et al., 2021).

**Figure 7:**
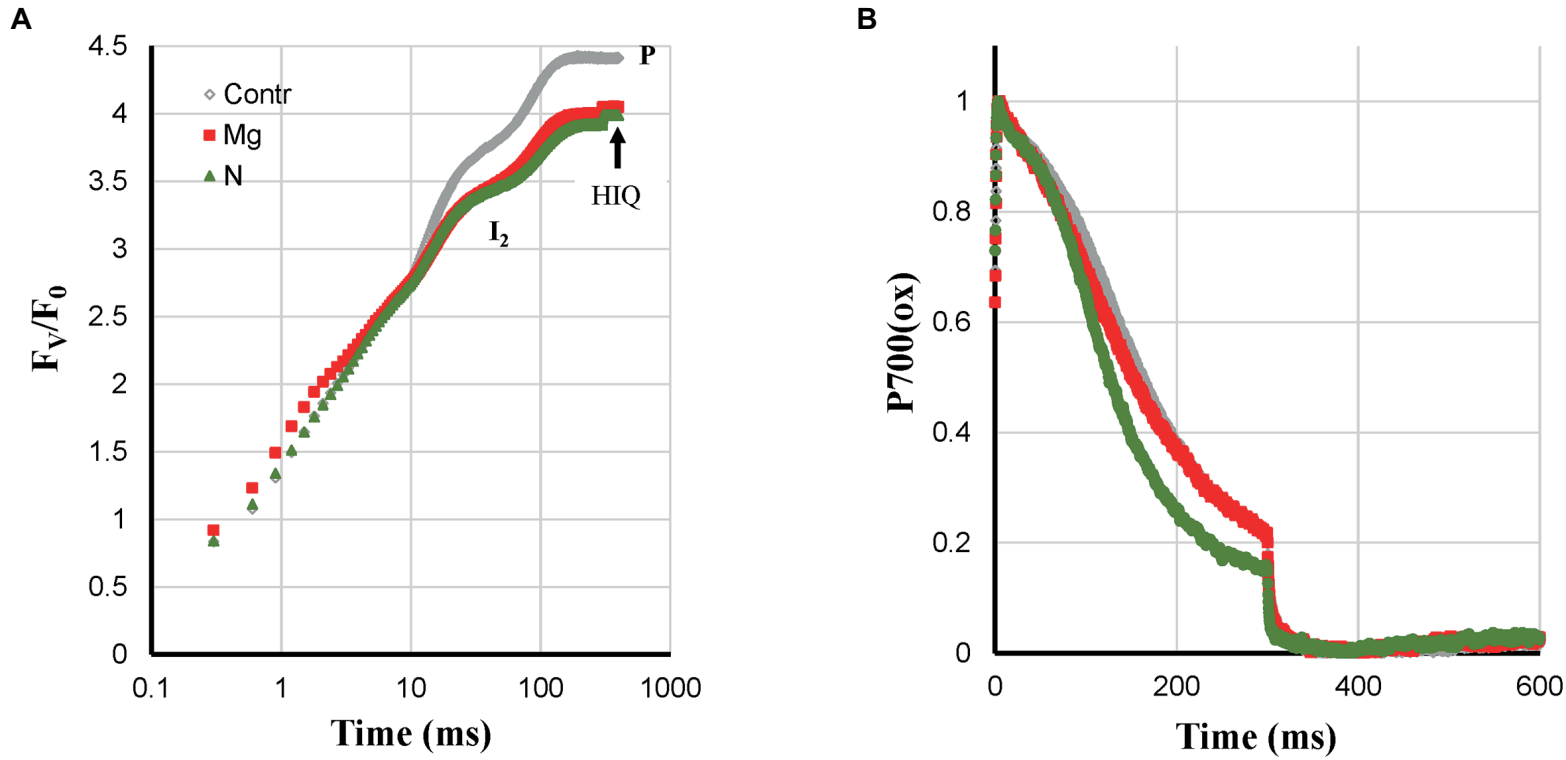
Effect of Mg- and N-deficiencies applied to hydroponically grown sunflower plants on the polyphasic rise **(A)** and P700^+^ **(B)** kinetics, respectively. The transients are averages of four independent measurements.

N-deficient plants showed a mix of deficiency-related changes. The quite strong Pm loss was also observed like for Ca-, K-, and Zn-deficiencies (Figure 1C). However, the associated fast P700-re-reduction was missing (Figure 7B), and this could be due to feedback inhibition. The N-deficient plants did show HIQ (Figure 7A).

The symptoms observed for Mg-deficient plants were a fractional Pm loss that was stronger than the I_2_-P (abs.) loss and there was nearly no effect on I_2_-P (rel.) compared to the Control, see Supplementary Figure 1. Increased ROS levels have also been described in the literature for Mg-deficiency, which could be responsible for the decrease of Pm. The absence of faster P700^+^ re-reduction kinetics (Figure 7B) could then be due to a counteracting effect of destacking induced by Mg-deficiency causing a mixed state as observed for N-deficiency.

For a general application of this data, more information on the development of the deficiency-related changes in each case would be needed, but the data show that it is possible to discriminate between the twelve deficiencies that were induced in these sunflower plants.

## Discussion

### Diagnosis of Mineral Deficiencies

As shown above, for the present dataset on 12 mineral deficiencies, there is enough kinetic information to separate all 12 of them. Mn-deficiency stands alone. Only Mn can be used for a functional Mn-cluster. The other 11 deficiencies can be assigned to 4 different classes, with N-deficiency possibly belonging to two different classes: 1. P- and Cu-deficiency affecting the electron flow between the PQ-pool and P700; 2. Ca-, K-, and Zn- (and N-) deficiency associated with damage to the PS I acceptor side and fast P700^+^ re-reduction kinetics; 3. Fe- and S-deficiency affecting the synthesis of FeS-clusters; and 4. Mo- and B- (and N-) deficiency leading to feedback inhibition. The Mg- and N-deficiencies are treated in a separate figure (Figure 7) representing mixed states; they show a loss of Pm, but the P700^+^ re-reduction kinetics are not faster than in the control. For these four classes, the challenge is to separate in each case the class-members. For each deficiency, there is one time point available. It is, therefore, not possible to judge at what point during the development of the different deficiencies it is possible to separate them. It would seem likely that during early stages of the mineral deficiency the effects will be too small or too aspecific for a precise diagnosis. However, more extensive research would be needed to determine this.

### Biological Variability Between Plant Species: Electron Flow From the PQ-Pool to P700

**Figure 8:**
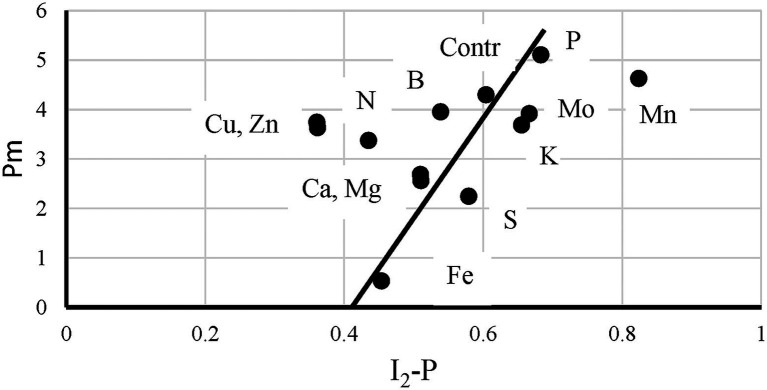
The relationship between the P700^+^(I_2_-P) and I_2_-P kinetics measured on leaves of control plants and of plants exposed to 12 mineral deficiencies. The points are averages of four independent measurements.

Although the data analyzed here, confirm observations made earlier for other plant species, the kinetic properties of sunflower leaves seem to differ from, for example, sugar beet leaves. In sugar beet leaves, a reasonable correlation between I_2_-P and the P700 + PC signal was observed (Ceppi et al., 2012). Here, a P700 loss did translate itself into a proportionally smaller I_2_-P amplitude, but with a considerable offset (see Figure 8 and “Correlation Between I_2_-P and Pm Amplitudes”). Possibly, the limitation posed by the cyt *b*_6_*f* complex in sunflower is considerably stronger than in sugar beet. This was supported by observations made on P-deficient plants. In the literature, it has been described for P-deficient barley and radish plants that this led to a loss of the I_2_-step (Frydenvang et al., 2015; Carstensen et al., 2018; Cetner et al., 2020). In sunflower, this was not observed. However, in the case of Fe- and S-deficient plants, it should also be considered that the Rieske protein of cyt *b*_6_*f* of sunflower could be more sensitive to these deficiencies than that of sugar beet.

### Interpretation of the Polyphasic Fluorescence Rise

Fluorescence induction curves: O-I_1_-I_2_-P or O-J-I-P are kinetically complex. And there is still no consensus on the role of the underlying processes. The redox state of Q_A_ is the dominant factor, but there are several other factors that may play a larger or smaller role. E.g., transient accumulation of P680^+^, especially when very high pulse-light-intensities are used (higher than 5,000 μMol photons m^−2^ s^−1^). Morin (1964) and Delosme (1967) discriminated between a photochemical phase reflecting the reduction of Q_A_ and a thermal phase reflecting another process sensitive to the temperature. With modern instruments, it is possible to quantify the photochemical phase very precisely by inducing the O-I_1_ rise by strong light, giving a single turnover flash after 1 ms to make sure that all Q_A_ is reduced. Although people have speculated about variable PS I fluorescence for many years there was no clear experimental evidence for its existence in leaves. Tóth et al. (2005) showed for several plant species that well dark-adapted leaves, subsequently passively infiltrated with DCMU, had F_M_-values that were independent of the redox state of PS I. Nearly identical F_M_-values were observed in either the presence or absence of DCMU. Two recent publications treat this topic experimentally in intact organisms (Pfündel, 2021; Schreiber and Klughammer, 2021). By measuring at wavelengths shorter than 710 nm and longer than 700 nm, respectively, a comparison could be made between induction curves reflecting mainly PS II emission and curves reflecting both PS II and PS I emission. Schreiber and Klughammer (2021) could clearly show a role of variable PS I fluorescence in green algae and cyanobacteria. In light green young *Hedera* leaves, they also detected variable PS I fluorescence, whereas Pfündel (2021) working with dark, mature *Prunus laurocerasus* leaves concluded that there was no variable PS I fluorescence. Analytically, a difference between both studies was that Schreiber and Klughammer (2021) normalized their data to the O-I_1_ amplitude, whereas Pfündel (2021) normalized his data on the variable fluorescence. However, could it be that biological variability between plant species as illustrated in the previous paragraph is a more important reason for this discrepancy? The sunflower data discussed here compared to observations made on barley and sugar beet could support effects of such biological variability.

### Do Sunflower Plants Show Variable PS I Fluorescence?

**Figure 9:**
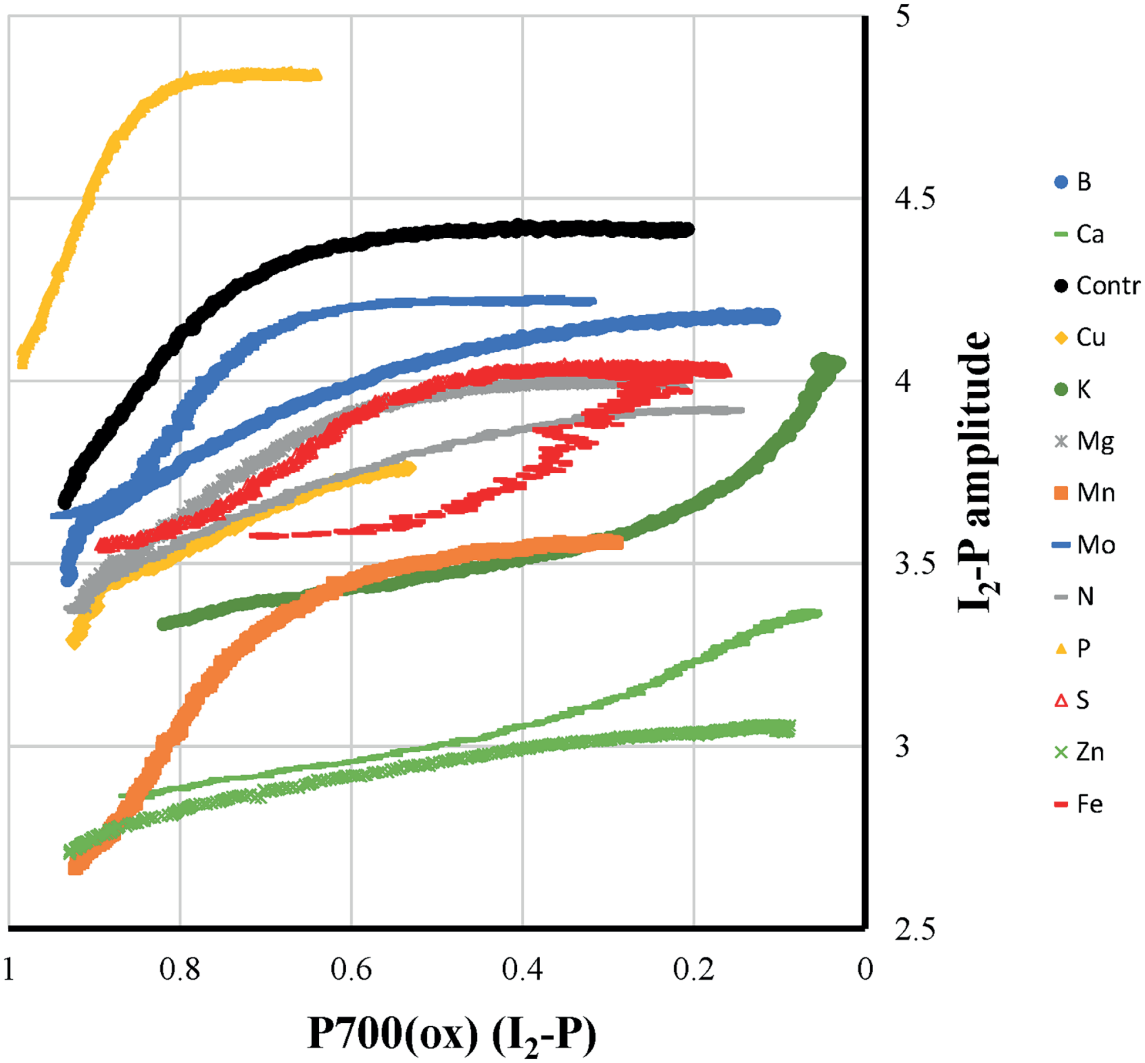
Relationship between Pm and I_2_-P amplitude for plants exposed to 12 mineral deficiencies. The colors reflect the different classes: yellow, electron transfer between cyt *b*_6_*f* and P700 affected; green, PS I acceptor side damage; red, FeS-cluster synthesis affected; blue, feedback inhibition; and gray, mixed states. Further, Mn-deficiency is orange, and the Control is black. The data represent averages of four independent measurements.

If variable PS I fluorescence exists in sunflower plants, we would expect it to develop during the I_2_-P phase. Only during this kinetic phase, a reduction of both the donor and acceptor side of PS II occurs, creating a PS II like state. If there is variable PS I fluorescence you would expect at the end of the PS I reduction phase a more or less linear correlation between the redox state of P700 and the fluorescence rise. In Figure 9, these two parameters are shown as a function of each other. For most of the mineral deficiencies, the relationship consists of a fast fluorescence increase in the beginning, where most P700 is still in the oxidized state, followed by no or a slow fluorescence increase while most of the P700^+^ is re-reduced. The casual observer may think that K-, Ca-, and Fe-deficiencies may form an exception. However, the upward curve of the relationship on going to full P700^+^ reduction is due to faster P700^+^ reduction kinetics without changes in the kinetics of the I_2_-P rise. In other words, this would point more toward the absence of a relationship between the two parameters. At the other extreme, we have P-deficient plants, where at 20% reduced P700, P is almost reached. Comparing the double-normalized I_2_-P kinetics of Control and P-deficient plants, they are essentially identical (Figure 2C). This strongly suggests that the I_2_-P rise is determined in sunflower by kinetic factors not directly related to the PS I and P700 redox state. This would support, at least for sunflower plants, the conclusions of Pfündel (2021).

### Correlation Between I_2_-P and Pm-Amplitudes

Ceppi et al. (2012) showed for S-deficiency that the 820 nm signal correlated with the I_2_-P amplitude, both relative and absolute, in sugar beet leaves. As shown by Çiçek et al. (2018) for chickpea, this correlation breaks down if electron transfer between the cyt *b*_6_*f* complex and P700 is inhibited by salt stress. In Figure 8, this relationship is checked for the sunflowers used in this study. Instead of the maximum P700 + PC signal, the Pm is plotted here as a function of I_2_-P (abs.). A trendline was drawn through the core set of points. The plot has two clear outliers. In Mn-deficient plants, PS II generates too few electrons to allow a saturation of the I_2_-step. As a consequence, the I_2_-P amplitude is proportionally larger than the I_2_-P amplitude of any of the other deficiencies (e.g., Supplementary Figure 1). On the other side, Cu is a clear outlier. The loss of PC has a kinetic effect on the I_2_-P amplitude, making it smaller. Zn is also an outlier, but in that case, it probably has to do with the loss of variable fluorescence (see Figure 4A). Finally, N also lies outside the trendline. As Figure 7A shows, N-deficient plants, like Cu-deficient plants, have relatively slow I_2_-P rise kinetics, although no clear reason for this feature can be provided. The trendline suggests that the “Pm is 0” line intersects the I_2_-P axis around 0.4. This could mean that not the whole I_2_-P phase is sensitive to the presence of PS I, or, which is maybe more likely, that the inflection point that is considered the I_2_ step overestimates the I_2_-P amplitude, because it still contains a considerable contribution of the I_1_-I_2_ phase. Or a mix of both. Ignoring this offset, though, there seems to be a rough correlation between I_2_-P and Pm in sunflower as well. However, comparing the sugar beet and sunflower data clearly illustrates the kinetic variability between plant species.

Another example of an experiment that was interpreted to show that there is no correlation between I_2_-P phase and PS I was published by Zivcak et al. (2015). These authors applied a series of strong 300 ms pulses spaced 10 s apart and assumed that their treatment predominantly inactivated PS I. The authors wrote that a decrease of F_V_/F_M_ from 0.78 to 0.72 equated an 8% decrease. However, as a simple calculation shows, at the same time, this 8% F_V_/F_M_ decrease equates an almost 28% decrease in F_V_. As was shown in several studies, O_2_-evolution extrapolates to zero around an F_V_/F_M_ of 0.4–0.44 (Van Wijk and Krause, 1991; Schansker and Van Rensen, 1999) and 0.06/0.38 would already equate a decrease of 16%. If we would assume that the maximum quantum yield of the control would equate the literature value of 0.84, the decrease would further increase to 27%. In other words, the damage done by the treatment to PS II was in this case considerably stronger than the authors assumed. As a consequence, the fact that they did not observe an effect of their treatment on the relative I_2_-P amplitude provides little experimental basis for their thesis that PS I activity did not affect the fluorescence rise. A chilling experiment could in this respect have provided the authors with a method to damage PS I more specifically.

### P-Deficiency, Inhibition of ATP-Synthase, and Photosynthetic Control

Carstensen et al. (2018) showed for P-deficient barley plants a drop in the stromal P-concentrations and suggested on that basis that P-deficiency led to inhibition of ATP-synthase. On the basis of the measurements discussed here, this effect cannot be measured directly. However, low ATP-synthase activity will slow down the export of protons from the lumen to the stroma and is, therefore, expected to lead to an accelerated acidification of the lumen, which in turn will accelerate the slowdown of the re-oxidation of plastoquinol by the cyt *b*_6_*f* complex. In other words, it will speed up the generation of Photosynthetic Control. This interpretation is confirmed by the slow P700^+^ re-reduction kinetics observed in Figure 2B. The fact that under these conditions, P is reached while still more than 60% of P700 is in the oxidized state (Figure 9) has important implications for our understanding of Chl *a* fluorescence measurements. Together with the PC-loss occurring in Cu-deficient plants (Ohnishi et al., 2021), this offers experimenters two very interesting model systems for the study of electron flow from the PQ-pool to PS I as well as processes like the xanthophyll cycle and NPQ-formation that are regulated by the lumen pH.

### Faster P700^+^ Re-Reduction in Ca-, K-, and Zn-Deficient Plants

Another new experimental observation is formed by the much faster P700^+^ re-reduction kinetics during the I_2_-P phase (Figure 4) observed for deficiencies that according to the literature cause increased ROS levels (Tainer et al., 1983; Bowler et al., 1992; Cakmak, 2005; De Freitas et al., 2016). These kinetics were associated with a Pm loss but not accompanied by faster I_2_-P rise kinetics. For the S-deficient plants, a stronger Pm loss was observed (Figure 5B) but there the P700^+^ re-reduction kinetics were considerably slower (Figure 5B). It suggests that the effect is not determined by PS I content. If the deficiency-induced ROS would lead to damage and inactivation of the FeS-clusters on the acceptor side of PS I it would strongly reduce the pool of electron acceptors available on the acceptor side of PS and this could explain the observed faster kinetics. That would mean that the faster kinetics are not a reflection of a loss of PS I but would then reflect the presence of PS I reaction centers with a damaged acceptor side. The data on N-, Mg-, and Fe-deficient plant suggest though that this ROS-induced effect may remain partially hidden if the deficiency causes other lesions: feedback inhibition in the case of N-deficiency, destacking in the case of Mg-deficiency, and a lower PS I synthesis in the case of Fe-deficiency.

### Signatures

An important goal in quite a few fluorescence studies is to define stress effects on the fluorescence kinetics that allow an unambiguous identification of different stresses. We had the same ambition here. Below a signature is defined for the twelve different deficiencies.

#### Mn-Deficiency

Slower fluorescence rise, large I_2_-P amplitude (lower F_V_/F_0_ ratio), and no effect on the Pm amplitude.

#### P-Deficiency

Like Cu-deficiency, slow and very incomplete P700^+^ re-reduction kinetics during the I_2_-P phase. Only deficiency that leads to higher F_V_/F_0_ ratio than in Control plants and I_2_-P rise kinetics that are nearly the same as in Control plants.

#### Cu-Deficiency

Like P-deficiency, slow and very incomplete P700^+^ re-reduction kinetics during the I_2_-P phase, however, these kinetics were more complex. Lower F_V_/F_0_ ratio than in Control plants and slower I_2_-P rise kinetics accompanied by a lag-phase.

#### S-Deficiency

Strong Pm loss (50% of Control), partially reduced PQ-pool in darkness, relatively strong HIQ signal, only slightly faster P700^+^ re-reduction kinetics during the I_2_-P phase.

#### Fe-Deficiency

Strong Pm loss (12% of Control), partially reduced PQ-pool in darkness, relatively strong HIQ signal, in contrast to S-deficient plants fast P700^+^ re-reduction kinetics during the I_2_-P phase.

#### Ca-Deficiency

Fast P700^+^ re-reduction kinetics during the I_2_-P phase, no PQ-pool reduction in darkness, small HIQ signal, Pm loss, but no effect on the relative I_2_-P amplitude, relative small F_V_/F_0_ ratio (3.35).

#### K-Deficiency

Fast P700^+^ re-reduction kinetics during the I_2_-P phase, no PQ-pool reduction in darkness, no HIQ signal, Pm loss, but no effect on the relative I_2_-P amplitude, relative large F_V_/F_0_ ratio (4.05).

#### Zn-Deficiency

Slightly slower P700^+^ re-reduction kinetics during the I_2_-P phase than observed for Ca- and K-deficient plants, no PQ-pool reduction in darkness, nearly no HIQ signal, Pm loss, but nearly no effect on the relative I_2_-P amplitude, small F_V_/F_0_ ratio (3.05).

#### Mg-Deficiency

No effect on the P700^+^ re-reduction kinetics during the I_2_-P phase, no PQ-pool reduction in darkness, small HIQ signal, fraction Pm loss > fraction I_2_-P (abs.) loss, no effect I_2_-P (rel.).

#### B-Deficiency

More sigmoidal P700^+^ re-reduction kinetics during the I_2_-P phase than in Control plants, no PQ-pool reduction in darkness, no HIQ signal, high Y(NA), and little Y(ND) during most of 10 min of actinic illumination.

#### Mo-Deficiency

Incomplete re-reduction of P700^+^ at P, partial reduction of the PQ-pool, small HIQ signal, no Pm loss and no effect on I_2_-P amplitude, high Y(NA), and little Y(ND) during most of 10 min of actinic illumination.

#### N-Deficiency

Only somewhat faster P700^+^ re-reduction kinetics during the I_2_-P phase than in Control plants, no PQ-pool reduction in darkness, relatively strong HIQ signal, Pm loss comparable to K- and Zn-deficiency. Fractional Pm, I_2_-P (abs.) and I_2_-P (rel.) loss were similar.

## Data Availability Statement

The raw data supporting the conclusions of this article will be made available by the authors, without undue reservation.

## Author Contributions

Reading the paper of Ohnishi et al. (2021) published in antioxidants, GS asked the authors if they were willing to share the fast kinetic data, they measured but did not use in their paper. On the basis of these measurements, GS made an analysis of the shared data and wrote the paper. With respect to the experimental research used for this analysis, CM conceived the research plan and was involved in the original analysis of the data. MO did most of the experiments. RF designed the experiments and was involved in the original analysis of the data. All authors contributed to the article and approved the submitted version.

## Funding

This work was supported by Core Research for Evolutional Science and Technology (CREST) of Japan Science and Technology Agency, Japan (grant number JPMJCR1503 to CM).

## Conflict of Interest

GS was employed by the company Heinz Walz GmbH.

The remaining authors declare that the research was conducted in the absence of any commercial or financial relationships that could be construed as a potential conflict of interest.

## Publisher’s Note

All claims expressed in this article are solely those of the authors and do not necessarily represent those of their affiliated organizations, or those of the publisher, the editors and the reviewers. Any product that may be evaluated in this article, or claim that may be made by its manufacturer, is not guaranteed or endorsed by the publisher.

## Supplementary Material

SUPPLEMENTARY FIGURE 1 The average values from Figure 1 were taken, normalized to the Control values, and shown in a single figure to allow an easy comparison of relative changes in the parameters Pm, I_2_-P (abs.), and I_2_-P (rel.).

## Abbreviations

F_0_, F_M_, and F_V_: minimum, maximum, and variable fluorescence
HIQ: high intensity quenching
O-I_1_-I_2_-P or OJIP: fluorescence rise between minimum and maximum fluorescence induced by high light intensity on a dark-to-light transition and defined by its intermediate steps
P700: PS I reaction center chlorophyll pair
PC: plastocyanin
Pm: maximum amplitude of the P700 signal (difference between all reduced and oxidized)
Q_A_ and Q_B_: primary and secondary quinone electron acceptors of PS II
Y(II) and Y(I): operational quantum yields of PS II and PS I, respectively
Y(ND) and Y(NA): quantum losses due to PS I donor and acceptor side limitations
