# Supplementary figures and images for: Identification of Twelve Different Mineral Deficiencies in Hydroponically Grown Sunflower Plants on the Basis of Short Measurements of the Fluorescence and P700 Oxidation/Reduction Kinetics
Source: Front Plant Sci. 2022 Jun 2;13:894607. doi: 10.3389/fpls.2022.894607 (PMC9201956; doi:10.3389/fpls.2022.894607)

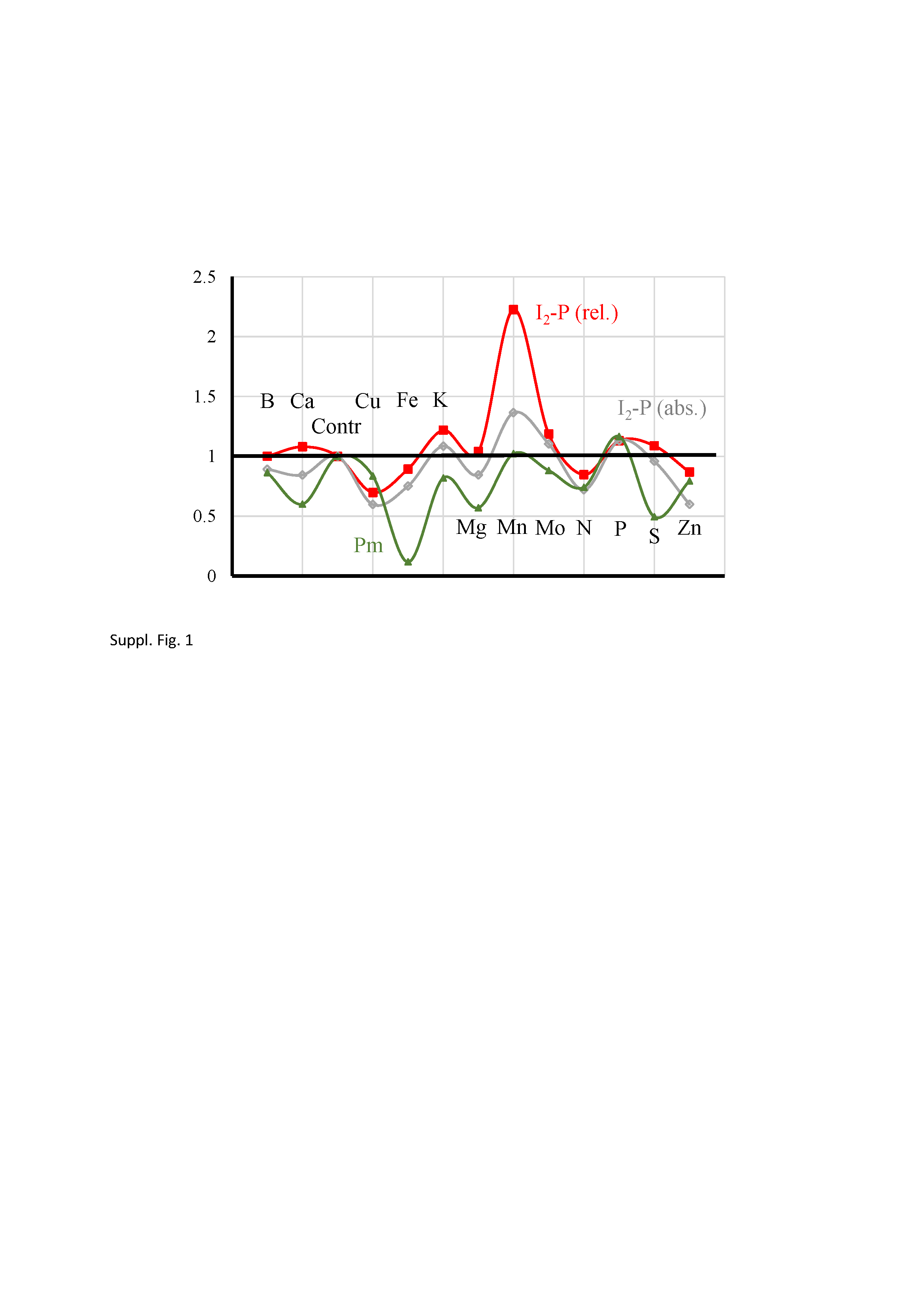

Supplement: SUPPLEMENTARY FIGURE 1 — The average values from Figure 1 were taken, normalized to the Control values, and shown in a single figure to allow an easy comparison of relative changes in the parameters Pm, I2-P (abs.), and I2-P (rel.). [file Image_1.tif]
